# Biogeography of Italy revisited: genetic lineages confirm major phylogeographic patterns and a pre-Pleistocene origin of its biota

**DOI:** 10.1186/s12983-021-00418-9

**Published:** 2021-06-29

**Authors:** Thomas Schmitt, Uwe Fritz, Massimo Delfino, Werner Ulrich, Jan Christian Habel

**Affiliations:** 1 Senckenberg Deutsches Entomologisches Institut, 15374 Müncheberg, Germany; 2 Department of Zoology, Institute of Biology, Faculty of Natural Sciences I, Martin Luther University Halle-Wittenberg, 06099 Halle (Saale), Germany; 3 Entomology and Biogeography, Institute of Biochemistry and Biology, Faculty of Science, University of Potsdam, 14476 Potsdam, Germany; 4 Museum of Zoology (Museum für Tierkunde), Senckenberg Natural History Collections Dresden, 01109 Dresden, Germany; 5 Dipartimento di Scienze della Terra, Università di Torino, Via Valperga Caluso 35, 10125 Torino, Italy; 6 Institut Català de Paleontologia Miquel Crusafont, Universitat Autònoma de Barcelona, Edifici ICTAICP, Carrer de les Columnes s/n, Campus de la UAB, 08193 Cerdanyola del Vallès, Barcelona, Spain; 7 Department of Ecology and Biogeography, Nicolaus Copernicus University, 87-100 Toruń, Poland; 8 Evolutionary Zoology, Department of Biosciences, University of Salzburg, 5020 Salzburg, Austria

**Keywords:** Pliocene, Quaternary cycles, Marine transgressions, Plate tectonics, Topography, Barrier, Refugia, Genetic structures, Phylogeography

## Abstract

Warm-adapted species survived the cold stages of the past glacial-interglacial cycles in southern European peninsulas and recolonized major parts of Central and Northern Europe in the wake of postglacial warming. However, many of the genetic lineages which differentiated within these refugia predate even the Pleistocene. One of these refugia is the Italian Peninsula with its surrounding islands. In this meta-analysis, we compiled phylogeographic patterns of multiple species across this region. We transformed existing geographic information on 78 animal and plant species (with a total of 471 genetic lineages) within 17 predefined areas into presence/absence matrices. We elaborated three matrices: (i) only old lineages exclusively distinguished by deep splits, (ii) only young lineages distinguished by shallow (i.e. recent) splits, and (iii) presence/absence of the respective species. To infer biogeographic relationships between the predefined areas, we performed bootstrapped neighbour joining cluster analyses on these three matrices. In addition, we reviewed the geological history of Italy to identify causes of the observed biogeographic patterns. We found Sardinia and Corsica to be biogeographically closely linked with each other, and that they diverge strongly from all other regions. Sicily also diverges strongly from all other regions, while the intra-island differentiation was comparatively low. On the Italian mainland, Calabria exhibited the most pronounced biogeographic differentiation, often with several lineages present, resulting from old vicariance events within the region. Furthermore, southern Apulia and the Po Plain with adjoining areas of northern peninsular Italy displayed considerable distinctiveness. Admixture prevailed in the areas between these three regions. The ancient isolation of Sicily, as well as Sardinia plus Corsica, resulted in endemic lineages with only moderate recent exchange with adjacent mainland regions. Pronounced diversification occurs within the Italian Peninsula. The complex tectonic activities, such as shifting (micro)plates, submergence of major parts of peninsular Italy with the genesis of numerous Pliocene islands, in combination with the climatic cycles during the Pleistocene have most likely generated the current biogeographic pattern of species. Immigrations from the Balkan Peninsula into northern Italy partly accounted for the distinctiveness of this region.

**Supplementary Information:**

The online version contains supplementary material available at 10.1186/s12983-021-00418-9.

## Introduction

The Mediterranean Basin is a renowned biodiversity hotspot, in which taxa evolved and survived the Pleistocene cold phases [1–3]. However, many species have a much longer history of permanency and evolution in this region, reaching far into the Neogene [4]. Within the Mediterranean region, Italy has long been acknowledged to be an independent Mediterranean sub-centre of differentiation and glacial persistence, which was later confirmed by molecular analyses highlighting the Italian Peninsula as one of the major southern European refugia [5–7]. However, when addressing the fine-scale biogeographic substructures of the Mediterranean refugia, the geographically larger sub-centres (such as the Maghreb, Iberia, the Balkan Peninsula and Anatolia) showed pronounced sub-structuring, the so-called refugia-within-refugia [8]. These structures are particularly pronounced in the southernmost parts of the larger sub-centres, supporting the idea that these were not homogeneous refugial areas but further sub-structured into an, often large, number of geographically small subunits in which distinct lineages evolved in geographic separation [8]. However, only little was known about the fine-scale biogeographic structures of Italy and it seemed to be less diverse [2]. It was therefore often assumed that the biogeographic differentiation within Italy, due to its limited geographic size, is in general simpler than that of the large sub-centres, for which much more data were available by that time [2] (Italy: 301,217 km^2^ [9]; compared to 583,254 km^2^ (Iberia); ca. 500,000 km^2^ (Balkan Peninsula); ca. 750,000 km^2^ (Anatolia); and the Mediterranean refugium in the Maghreb is at least as large as Anatolia).

Recent studies on the phylogeography of Italy have now revealed a much more complex phylogeographic structuring than expected from its limited geographic size [3]. Many of these patterns predate the Pleistocene, reflecting divergences correlated with old geological processes, like a “museum of the Neogene” in which old phylogeographic lineages are preserved. Since the review by Schmitt in 2007 [2], a wealth of new data has become available, allowing a comprehensive meta-analysis of the biogeography of Italy with its surrounding islands. Below, we examine these data in the context of the complex geological and geographic history of the region, which is first briefly summarised to make it more understandable.

### The geological and geographic history of Italy

Due to the northwards drift of the African plate and the shrinking of the Tethys Sea, Italy has undergone quite complex geological transformations during the Tertiary [10]. In this process, the succession and intertwining of several geological phenomena was involved. Among others, these include the diachronic genesis of the Alps and Apennines, various eustatic events which repeatedly caused significant shifts of the coastline, and the rotation of blocks that ultimately led to the genesis of Corsica, Sardinia, as well as part of Sicily and Calabria. The biogeographically most relevant geological events are therefore briefly outlined.

The Alpine orogeny is an effect of the closure of the Tethys Sea after the collision of Adria (a northern African peninsula) and Europe. It started already in the Middle Cretaceous and is still on-going [11, 12]. It is likely that the Alps had at least partly emerged already in the Cretaceous, but the collision and hence their significant elevation occurred much later, during the Eocene. It has been estimated that from the Oligocene until today, the Alps were uplifted by about 1 mm/year [12], but Nocquet et al. [13] recently calculated that the actual uplift reaches ~ 2.5 mm/year in the north-western Alps. It is due to erosion processes that the Alps are not now much higher. Consequently, the current Alpine landscapes are mostly the product of the glacial events during the last few million years [12].

**Fig. 1:**
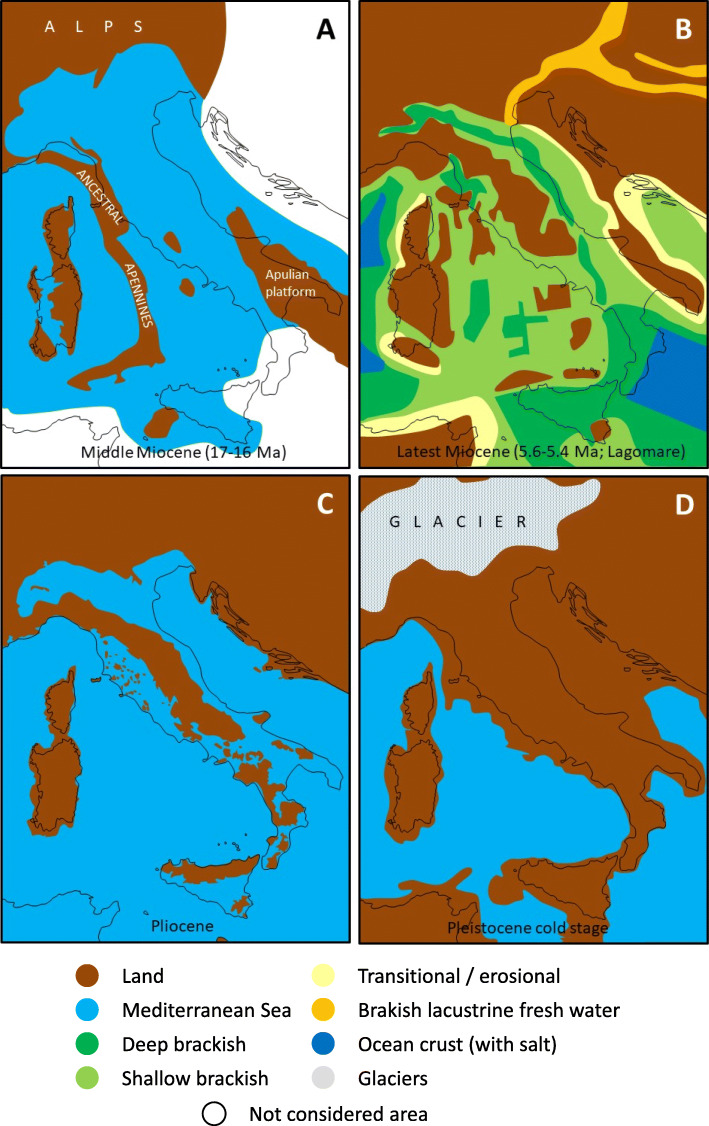
The changeful history of Italy and its surrounding regions from the mid-Miocene to the Pleistocene. (**a**) Mid-Miocene (17–16 Ma, the white areas are excluded, black lines show the recent situation), (**b**) the ‘Lago-Mare’ period (i.e., the first Messinian cycle or the lower gypsum mega-sequence), latest Miocene (6.0–5.6 Ma), (**c**) Pliocene (5.3–2.6 Ma), (**d**) Last Glacial Maximum (21 ka), similar distributions of land and sea are also assumed for the previous ice ages of the late Pleistocene. Maps based on Bosellini [12] and Vai [17]

The origin of Sardinia, Corsica, the southernmost tip of Calabria and the Monti Peloritani area in north-eastern Sicily is linked to the rotation of a block formerly located in the western Mediterranean sector. According to Advokaat et al. [14], the Corso-Sardinian block experienced a counter-clockwise rotation of about 45° already in the Eocene, followed by a further rotation of about 50°, mostly during the early Miocene, being much better documented than the first [15]. This overall rotation of about 95° shifted the block from the southern edge of the European continent to its current position and was essentially completed by 15 Ma (Fig. 1a). While the rotation of the Corso-Sardinian block is intensively investigated, the rotation of the precursors of the southernmost tip of Calabria (from the Piana di Sibari to the strait of Messina) and the Monti Peloritani in north-eastern Sicily has attracted much less attention. However, the geology of this area is the result of a mixture of events that span about 60 Ma and is among the most complex of the Italian Peninsula. These two “now southern” blocks followed the others, but their rotation amplitude was much greater. They rotated along with the Corso-Sardinian block until about 15 Ma (Fig. 1a), but then kept rotating counter-clockwise during the following 8 Ma, while the Tyrrhenian Basin opened [12]. The Calabrian–Peloritanian orogeny is therefore an arcuate segment of the peri-Mediterranean orogenic Alpine nappe system [16].

The origin of the Apennines is linked to the rotation of the Corso-Sardinian block and the opening of the Tyrrhenian Sea. About 15 Ma, when the Corso-Sardinian block reached its final position, the proto-Apennine chain was only partly formed and exposed above sea level. The northern proto-Apennine chain was already in a position close to the one currently occupied by Tuscany, whereas the southern proto-Apennine chain was located where the Tyrrhenian Sea is located today (Fig. 1a; [12]). The following counter-clockwise rotation of the proto-Apennine chain and its final structuring in the chain of today, occurred in the last 8 Ma as a consequence of the opening of the Tyrrhenian Sea, the youngest and among the deepest (about 3600 m) of the basins of the Mediterranean Sea [12].

Sea level fluctuations certainly impacted massively the dispersal of terrestrial organisms and, therefore, gene flow. Three episodes could be particularly relevant for the understanding of the extant associations: the Messinian Salinity Crisis (MSC; Fig. 1b), the Pliocene transgression (Fig. 1c), and the glacial regressions that most recently culminated with the Last Glacial Maximum (LGM; Fig. 1d). The best studied, but still not fully understood event is the MSC, which occurred between 5.97 and 5.33 Ma ago [18]. Major parts of the Mediterranean Sea quickly dried out, leaving an arid landscape similar to intermontane deserts of California and Nevada [12, 19], interspersed during stage 3 with non-marine palaeobiotopes, i.e., a brackish lake system inhabited by ostracods, molluscs and dinocysts, the so-called ‘Lago-Mare’ (Fig. 1b; see Andreetto et al. [20], and references therein). However, new evidence from the Italian fossil record indicates that fish assemblages were dominated by taxa characterized by a strict marine affinity, thereby suggesting that, at least around the Italian Peninsula, the Mediterranean was filled by marine waters with a normal salinity [20]. In any case, it is likely that the paleo-environments and the paleogeography of some portions of the circum-Mediterranean area were significantly modified during the MSC. This period ended at about 5.33 Ma with the Zanclean flood, the opening of the Gibraltar Strait, and the rapid refilling of the Mediterranean Basin with Atlantic waters. According to some interpretations of the geological evidence, this may have involved peak rates of sea level rise in the Mediterranean of more than ten metres per day, and it has been estimated that 90 % of the water was transferred in a short period of time ranging from a few months to two years [21].

The palaeogeographic maps of the Pliocene Italian Peninsula show a reduced area of dry land compared to the present (Fig. 1c). A combination of factors caused this, including differences in the height of the mountain chains, tectonic activity, and eustatic changes. This, for example, resulted in a repeated transformation of southern Calabria into a chain of islands. After a moderate rise estimated to have reached about 20 to 25 m during the early Pliocene, the sea level fluctuated repeatedly, showing however a general decrease matching a general decline in global temperatures, and finally reaching the current level [22].

Because of these sea level oscillations, the extent of the coastal plains changed considerably throughout time, including the largest plain in Italy: the Po Plain. This plain belongs almost entirely to the Apennine foredeep, and extremely thick Pliocene and Quaternary sediments lie under the present-day surface [23]. Along with part of the bottom of the Adriatic Sea, the plain is covered by sediment eroded from the Alps. Its height ranges from 150 m a.s.l. in its western part to areas below sea level in the east [12]. During the Pliocene transgressions it was basically non-existent, because it was replaced by a relatively deep gulf when the Adriatic Sea reached the western edge of the plain, but later on, during the LGM (from 26.5 ka to about 20 ka; [24]), when the sea level dropped by about 130 m [22], it stretched in a south-easterly direction, reaching the latitude of Ancona or even southwards in the direction of the Gargano Promontory (Fig. 1d; [12, 25]), i.e., opening a broad connection to the western Balkan Peninsula.

The connections of the islands to the mainland are difficult to assess using a geologic basis that goes beyond the evaluation of sea level variations. Therefore, the detection of biogeographic affinities is usually involved in the study of these connections. As summarized by Masini et al. [26], the Italian fossil record provides information about three areas that are or were islands in the past: the Gargano paleo-archipelago, the Sardinia–Maritime Tuscany paleo-bioprovince, and the Sicilian insular complex. During the late Miocene–early Pliocene, Gargano was part of an archipelago whose faunal assemblages had a polyphasic origin due to different dispersal events. Worth noting is the presence of the same endemic taxa in Gargano and Scontrone (Abruzzi), a slightly older locality that is currently more than 110 km west of the fossiliferous localities of Gargano. The Apulian platform was connected to the Apennine chain during the early Pleistocene.

The late Miocene Tusco-Sardinian palaeo-bioprovince, including at least northern Sardinia and southern Tuscany, was unequivocally characterized by the presence of the same endemic mammalian taxa, indicating geographic proximity, or some kind of connection (see Abbazzi et al. [27], with references therein). Since the mid-Pleistocene, populations of various mammals were isolated as Sardinia became an island, and these subsequently differentiated into endemic taxa. Their assemblages displayed considerable stability, in contrast to the changes that occurred on the mainland [26].

### Towards a comprehensive understanding of the phylogeography of Italy

To comprehensively understand the phylogeographic structure of Italy (including Corsica), we analysed 90 phylogeographic studies based on different molecular markers (i.e., mt, cp, and nuDNA sequences; allozyme polymorphisms; microsatellites; others) and various taxa (including 54 vertebrate, 12 invertebrate, and 12 plant species). Note that these species are mostly terrestrial, and although some semi-aquatic taxa are also included, available data is still insufficient to draw clear conclusions on the biogeography of the freshwater systems of Italy. Based on these data, we identify the geographic locations of the centres of differentiation (i.e., the refugial areas where populations were isolated and consequently diversified into genetic lineages) and the paleo-geographic events which were responsible. We dissected Italy into 17 predefined geographic areas, according to well established geological (e.g. microplates, mountain ranges) and landscape structures (e.g. [28–31]). Against the background of the geological history of the Pliocene and Pleistocene range shifts, we ask the following three questions:

1. Do the islands of Sardinia plus Corsica as well as Sicily correspond to biogeographical sub-regions in comparison to the Italian Peninsula?
2. Do the biogeographic patterns revealed by our analyses correlate with geological microplates and islands, and are they compatible with what is known about the geological history of the Neogene?
3. Do sub-refugia exist within the Italian peninsular refugium? And if so, where are these located?

## Materials and methods

### Study region

**Fig. 2:**
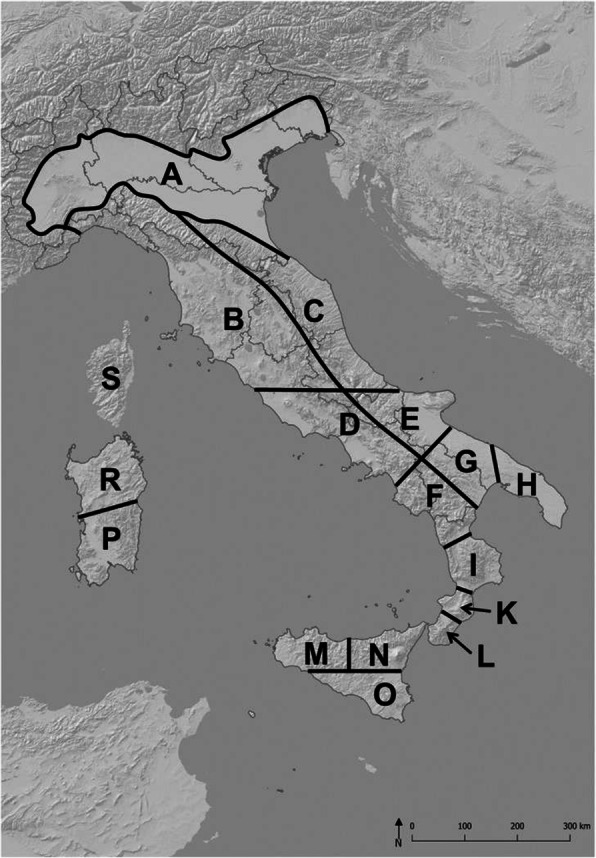
Overview of 17 predefined areas across Italy: A: Po Plain; B: Ligurian and northern Tyrrhenian area; C: central Adriatic, D: central Tyrrhenian area, E: Tavoliere and Gargano (and surrounding areas); F: southern Tyrrhenian area, G: Murge (and surrounding areas); H: Salentine peninsula; I: Sila; K: Serre; L: Aspromonte; M: northwestern Sicily; N: northeastern Sicily; O: southern Sicily; P: southern Sardinia; R: northern Sardinia; S: Corsica. Predefined areas use mountain ranges as divides, but their pattern is also more fine-grained in regions that should be more relevant for the diversification of a species into different genetic lineages due to their geologic history (see text)

We considered all major parts of Italy, but excluded the Alpine areas, because these represent a biogeographic entity very different from the rest of Italy [32, 33]. Since Corsica (politically part of France) is known to be biogeographically closely linked to Sardinia [34, 35], they were both included in the present study (Fig. 2). According to orography and established geographic provinces (e.g. [28–31]), the following 17 areas were distinguished (from north to south): A: Po Plain; B: Ligurian and northern Tyrrhenian area; C: central Adriatic, D: central Tyrrhenian area, E: Tavoliere and Gargano (and surrounding areas); F: southern Tyrrhenian area, G: Murge (and surrounding areas); H: Salentine peninsula; I: Sila; K: Serre; L: Aspromonte; M: north-western Sicily; N: north-eastern Sicily; O: southern Sicily; P: southern Sardinia; R: northern Sardinia; and S: Corsica. These areas are of different sizes, reflecting the different levels of geographic complexity found throughout Italy. Their resolution into smaller units is higher in regions that, due to their geological history (see above), are expected to be centres of diversification of species into different genetic lineages. The large islands Sicily and Sardinia were also divided into three and two areas, respectively, to assess possible intra-island differentiation; Corsica was not subdivided, due to its smaller size. However, size was not the sole reason for doing so. As Sicily was composed of several parts during the Earth’s history (see above), this subdivision makes phylogeographic signals resulting from this detectable; additionally, the western parts have the closest links to North Africa. On the other hand, Corsica is a relatively uniform block of acid bedrock forming one high mountain area surrounded by limited areas at lower altitudes. The landscape structures and geology of Sardinia, like Sicily, are much more complex, so that splitting it in two parts (i.e. northern and southern) seemed desirable.

### Biogeographic data

We evaluated 127 articles (published up to the end of February 2021) addressing biogeography and population genetics across major parts of peninsular Italy (including the Po Plain) and/or the three major adjacent islands (Corsica, Sardinia, and Sicily). Ninety of these articles contained data suitable for our analysis, i.e., data for several of the predefined areas, based on different molecular marker systems (i.e., mitochondrial (56), nuclear (10) and plastid (11) DNA sequences, microsatellites (8), allozyme polymorphisms (6), AFLPs (1), ISSR (1), chromosome number polymorphisms (1); note that one genetic data set has sometimes been derived from more than one article, and that more than one data set may have been presented within one article. An overview of all studies is provided in Appendix S1. We further assigned all studies to one of the following taxonomic categories: vertebrates (54 species), invertebrates (12), and plants (12). In total, we analysed phylogeographic data sets of 78 species representing 471 different genetic lineages from a total of 238 molecular markers, across their Italian range.

For each species, we assessed whether each genetic lineage is present or absent in each of our 17 predefined areas. In many cases, genetic information was not available for the entire distribution range in Italy and Corsica. If so, for an individual species, areas without genetic data were either considered ambiguous or assigned to the lineage occurring in the surrounding areas, if this was unequivocally possible (i.e., the respective lineage was present in all adjoining areas). Ambiguity was assigned to a lineage within an area without a record (1) if the adjoining areas had different genetic lineages (or even ambiguities) and a decision could not be made whether the respective lineage is present or not, or (2) the area without record is at the margin of the species distribution in Italy so that either the lineage of an adjoining area is present, or another still unknown lineage. This, in particular, affected the islands Sardinia and Sicily: if genetic information was not available for each of their, respectively, two and three areas, the area(s) without data was/were considered ambiguous, although the presence of this lineage on the entire island is likely. Areas with unsupported presence of a genetic lineage entered the statistical analyses as absence to avoid artificially increased biogeographic distributions and decreased discrimination power. Note that two or more lineages can co-occur within one single area. In cases in which one such lineages only marginally entered an area, this lineage was also recorded as present.

In addition, we assigned lineages to two age categories in cases where genetic structuring within a species showed a clearly hierarchical age structure and thus enabled an unequivocal distinction:

(i) Deeply divergent lineages (considering exclusively the deepest level of diversification in the data set and thus reflecting more ancient processes, i.e., representing old lineages), resulting in 213 units, and.
(ii) younger genetic lineages (corresponding only to the shallow genetic divergences and hence reflecting more recent processes; note that young genetic lineages are often nested in the old ones, i.e., one old genetic lineage can comprise two or more young lineages), resulting in 258 units.

If no such hierarchical structure was detected, no distinction into old and young lineages was performed. In these cases, the derived lineages were included in the analysis of the old genetic lineages. As molecular clocks are missing for many of the analysed datasets and the heterogeneity of the existing ones did not allow the implementation of *a posteriori* assignments of molecular clocks to all of them, this differentiation into old and young lineages merely reflects the hierarchical genetic structuring within the respective taxon. Therefore, these two sets of genetic lineages cannot be unequivocally assigned to two absolute time windows. It is only a relative ranking. Nevertheless, the biogeographic structuring revealed by analysis of the set of old lineages, separated by deep genetic splits, should be more ancient than the structures revealed by the analyses of the geographic distribution of the young lineages, distinguished only by shallow splits. Consequently, no clear dating of the structures was possible in our analyses. However, in cases where phylogeographic analyses have utilised molecular clocks, diversification events separating old lineages have mostly been assigned to the Pliocene, whereas Pleistocene age was often assigned to the diversification events involving young lineages. As a third matrix, we also used a presence/absence data set at the level of species. Additionally, we performed our analyses separately for vertebrates and plants; the amount of data for invertebrates was insufficient for such analyses.

### Statistics

For the two divergence levels (i.e., old and young genetic lineages) and the presence/absence data of species, we constructed three lineage × area presence-absence matrices. Therefore, our study is basically a phenetic approach, similar to a co-occurrence analysis in community ecology [36]. Consequently, our approach is not molecular in a narrow sense. For each of these three matrices, we calculated differently rooted neighbour joining cluster trees (unrooted, rooted in the Po Plain, and rooted in southern Sicily) based on the Sørensen index of compositional dissimilarity using Past 4.01 [37]. Rooting in the Po Plain and Sicily was used to better infer the possible latitudinal gradients in the biogeographic patterns. Given that Sicily and southern Italy served as major glacial refuges, we examined whether the existence of such refuges might be supported by respective latitudinal gradients of postglacial colonisation. In phenetic analyses, neighbour joining is still the best approach if the data structure requires the use of a dissimilarity metric, as in the present case. This approach is least sensitive to the specific underlying evolutionary processes and robust with respect to deviations from branching additivity [38]. We tested the reliability of the tree topology using comparisons from the matrix with 1000 bootstrap samples. Note that our data structure based on presences and absences did not allow for the application of maximum likelihood methods to construct the lineage split trees. Parsimony analysis performed with the present presence–absence data set returned highly inconsistent results. Numbers of lineages per area and the respective dissimilarity matrices are presented in Appendix S2.

We further calculated the pattern of spatial co-occurrence of each species at each level based on Sørensen dissimilarities *J* using the *Turnover* software [36]. As the Sørensen score depends on the total numbers of occurrences and areas, direct comparisons of raw scores might be misleading. Therefore, we applied a null model approach and compared observed scores with those obtained from 1000 equiprobable reshufflings of focal lineages across the 17 areas. We used equiprobable reshuffling because there is no *a priori* reason to expect that certain lineages cannot occur in any one of the Italian areas. From these null models, we calculated for each species *i* standardised effect sizes ${SES}_{i}=\frac{\varDelta {J}_{i}}{{\sigma }_{i}}$; where ΔJ_i_ is the difference between observed and expected J_i_, and σ_i_ is the standard deviation of the null model distribution. Under the assumption that this distribution is approximately normal, SES values > |2.00| indicate statistical significance at the two-sided 5 % error level.

## Results

**Fig. 3:**
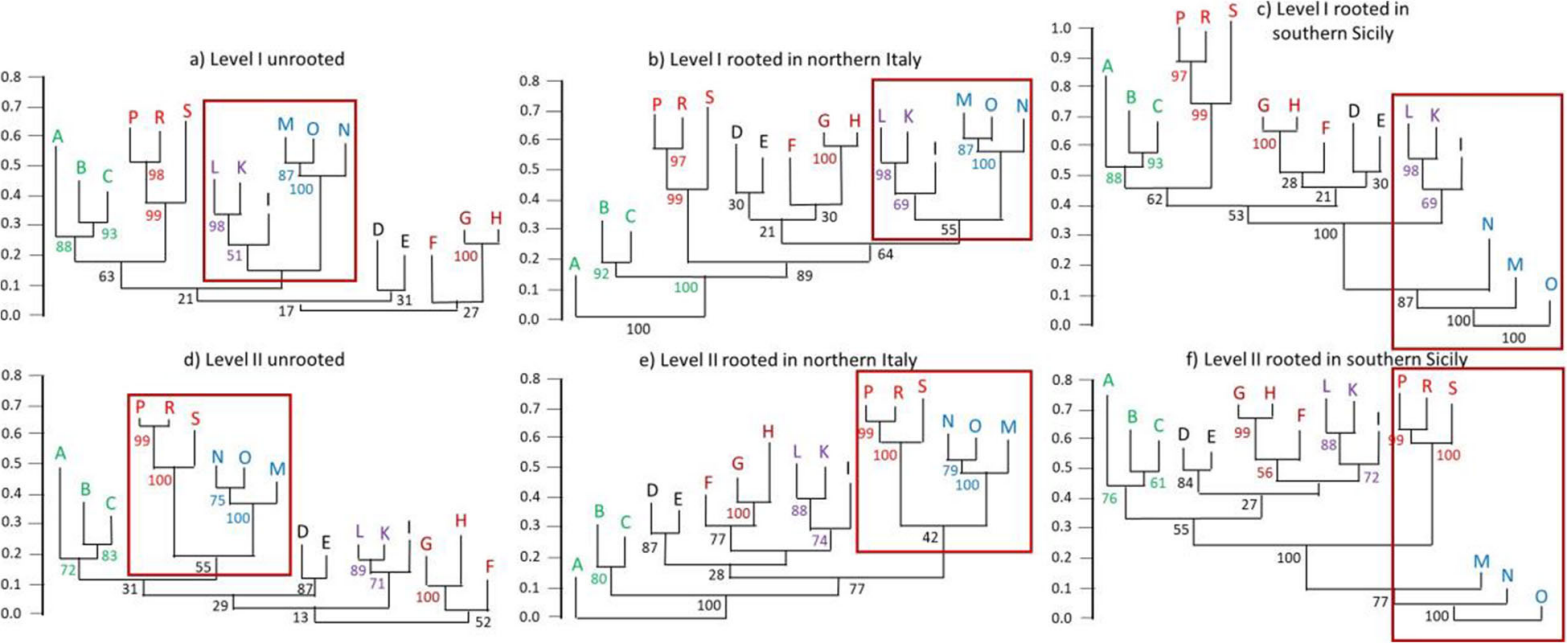
Neighbour joining cluster analyses (Sørensen dissimilarities) based on the occurrence of 471 genetic lineages. We used deeply divergent (i.e. old) genetic lineages (a–c; level I including 213 lineages) and such distinguished by shallow differentiations (i.e., referring to a younger time frame) (d–f, level II representing 258 lineages) for 78 species among 17 areas (A–S) as defined in Fig. 2. Trees in a and d are unrooted; in b and e rooted in the Po Plain; in c and f rooted in southern Sicily. Numbers show the percentage of bootstrapped trees (1,000 replicates) corroborating the focal split. Colours link areas consistently clustering together. The dark red square is highlighting the closest link of Sicily (M, N, O)

**Fig. 4:**
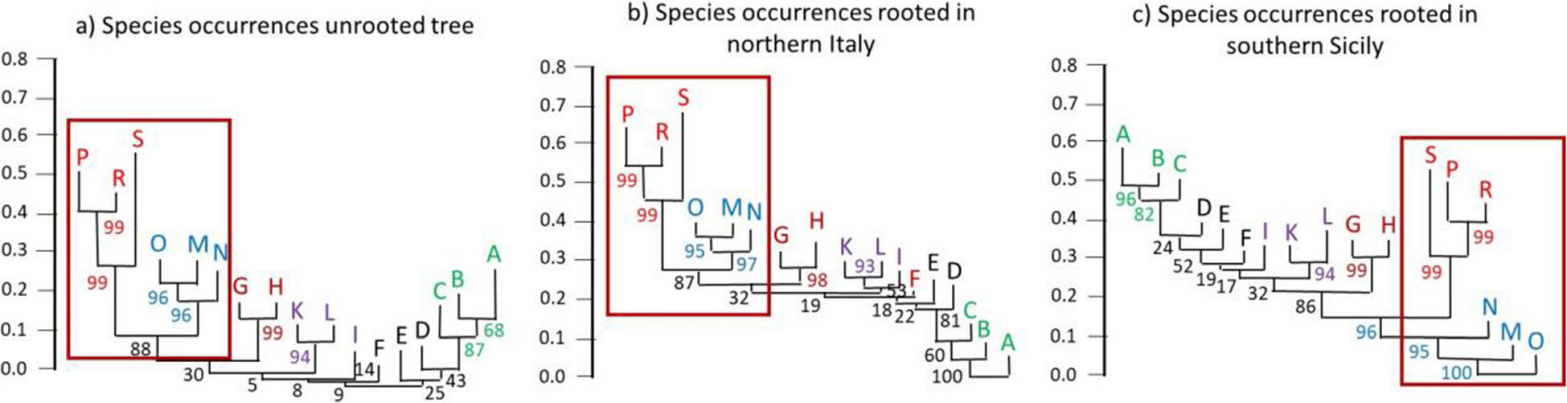
Neighbour joining cluster analyses (Sørensen dissimilarities) based on the occurrence of 78 species within 17 areas (A–S) as defined in Fig. 2. Trees are unrooted (a), rooted in the Po Plain (b), and in southern Sicily (c). Numbers show the percentage of bootstrapped trees (1,000 replicates) corroborating the focal split. Colours link sections consistently clustering together. The dark red square is highlighting the closest link of Sicily (M, N, O), which consistently is Sardinia and Corsica (P, R, S). The order of areas best matches the latitudinal mainland gradient

Neighbour joining cluster analyses (Figs. 3 and 4) detected, with significant statistical corroboration, major biogeographic structuring with segregation of several congruent geographic groups across the different genetic lineages and taxa. In all analyses, Sardinia and Corsica form one biogeographic unit, but with each island displaying pronounced differentiation. If the old lineages are considered only, these two islands appear to be loosely connected to northern Italy (Fig. 3a-c, level I). Sicily (M, N, O) represents a well-supported region of its own with little internal structure in all analyses. At the old lineage level, Sicily is consistently most closely linked to Calabria, but with low statistical support. Mainland Italy is divided into three regions: northern Italy (A–C, i.e., to the latitude of Rome) and two southern regions, i.e., Calabria (I, K, L) and Apulia (G, H). For the northern Italy group, the Po Plain is somewhat more differentiated from the two adjoining peninsular areas B and C than these among each other, although located on different sides of the Apennine chain (Fig. 3). Apulia does not cluster close to Sicily (Fig. 3, level I). The south-central Italian areas D, E and F do not consistently cluster with the regions of northern or southern Italy, but the southern Tyrrhenian area (F) mostly clusters close to Apulia. Rooting the tree in southern Sicily improves the discriminatory power of the tree and separates this island from the rest of Italy (Fig. 3c), with Sardinia and Corsica again recovered as a single distinct cluster close to northern Italy.

In turn, the younger lineages return consistent links of Sardinia and Corsica with Sicily (Fig. 3d-f). Nevertheless, Sicily as well as Sardinia with Corsica again appear as distinct clusters. Analyses of the younger lineages also retain Apulia and Calabria as distinct genetic clusters (Fig. 3d-f). Again, the southern Tyrrhenian area F clusters close to Apulia. The Po Plain remains somewhat more distinguished from the Ligurian and northern Tyrrhenian area B and the central Adriatic area C than these from each other (Fig. 3d-f).

Analyses restricted to vertebrates yield a pattern similar to that obtained for all taxa, but the Po Plain is more isolated in the former (Appendix S3 Fig. A1). At both levels, vertebrates of Corsica and Sardinia cluster closely to Sicily (Fig. A1). A separate analysis for the plant taxa (older lineages only) yielded a strong differentiation between Sicily and Calabria on the one hand and the rest of Italy on the other (Fig. A2). Furthermore, Corsica clusters with northern Italy, while Sardinia shows closer affinities to Apulia (Fig. A2).

The biogeographic pattern revealed for species occurrences resembles that of the younger genetic lineages (Fig. 4). An unrooted neighbour joining tree supports the similarity of Sardinia and Corsica (Fig. 4a), with Sicily clustering closest but with remarkable distinction. Despite the geographic proximity of southern Calabria and Sicily, our data do not reveal a consistent clustering of both regions and thus no close biogeographic cohesion. Northern Italy (A–C) is supported as a distinct unit. Rooting of the occurrence tree in the Po Plain results in a clear north–south gradient of species occurrences (Fig. 4b). The strongest discriminatory power has the tree rooted in southern Sicily (Fig. 4c) clearly indicating the distinctiveness of Sardinia and Corsica, Sicily, as well as of southern Calabria (K, L). Also, Apulia (G, H) and northern Italy with the Po Plain (A–C) appear to be distinct biogeographic units. Mantel correlations of Sørensen dissimilarities based on distribution patterns of genetic lineages and species recover high correlations for the deeply divergent lineages (*r* = 0.82, permutation *P* < 0.0001) and a weaker correlation for the younger lineages (*r* = 0.57, *P* < 0.0001).

**Fig. 5:**
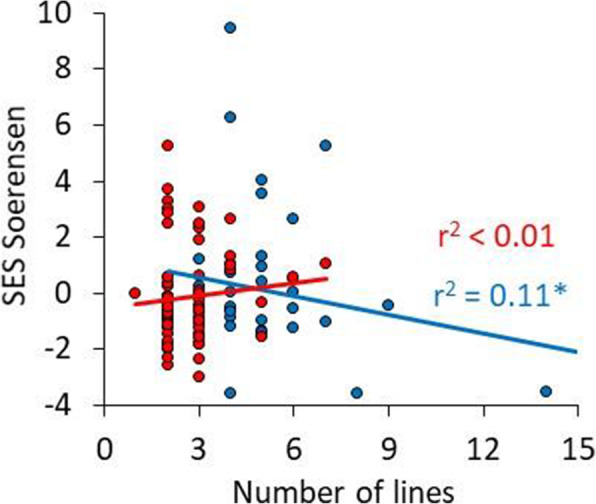
Standardized effect sizes *SES* of the Sørensen index of dissimilarity in spatial co-occurrence between genetic lineages of all species studied in dependence of the species-specific number of lineages. Red dots refer to the deeply divergent (i.e. old) lineages, blue to younger lineages. Permutation significance of the blue regression line: *: P = 0.03)

In our species-specific approach, we look at the pattern of co-occurrence of all species (Fig. 5). Fifty-two of the 77 species with data at the old divergence level (i.e. 67.5 %) have positive Sørensen dissimilarities across the 17 regions, while 25 of the 39 species for which information on younger lineages is available (64.1 %) have positive SES scores (Fig. 5), indicating a tendency towards spatial lineage segregation. In the younger lineages, spatial segregation decreases with increasing numbers of species occurrences (Fig. 5).

## Discussion

As an answer to our first research question about the biogeographic importance of the three large islands adjoining peninsular Italy, our meta-analysis strongly suggests that Sardinia plus Corsica as well as Sicily represent the two most divergent biogeographic units. Both have some similarity when only shallow genetic divergences are considered but not for the lineages distinguished by deep divergences (i.e., reflecting the old level of differentiation). Mainland Italy also shows a strong biogeographic structuring. Southern Calabria (K, L), Apulia (G, H) as well as the northern half of peninsular Italy (to the latitude of Rome, areas B and C, including the Po Plain, A) consistently cluster together and correspond to three distinct biogeographic units. Northern Calabria (I) is part of the Calabrian region but is distinguished remarkably from southern Calabria (K, L) in all analyses. The remaining areas in southern central and southern Italy (i.e., D, E, F) constitute a transition zone between the two southern Italian regions on the one hand and northern Italy on the other. Consequently, we confirm the hypotheses implicit in our second initial question, that the biogeographic structures correlate with the geological history of Italy and its surrounding islands in the Neogene. Below, we discuss in detail this relationship between genetic structures and the geological, orographic and environmental history.

The biogeographic uniqueness of Sardinia and Corsica is easily explained by their geological history [39]. Both islands are part of the Tyrrhenian microplate and detached from the eastern Iberian Peninsula via a counter-clockwise rotation of their microplate [14], allowing for a long-standing isolation that explains the high amount of old endemic lineages and the high degree of endemic species [40]. The high cohesiveness between the two islands is not only explained by their shared long-term history, but also by repeated connections during periods of glacial low sea levels (Fig. 1d-f, [39, 41]). For the older lineages, a signal for a relationship between Sardinia plus Corsica and northern peninsular Italy was detected. This reflects the geographic proximity of these two regions, but is also the result of relatively recent (most likely Pleistocene) immigrations from the mainland, facilitated by its connection to Corsica and Sardinia via the Tuscan Archipelago during glacial periods of low sea levels [42]. This has been shown, for instance, for one lineage of the Italian wall lizard *Podarcis sicula* [43, 44] and the hedgehog *Erinaceus europaeus* [45]. However, care is required in the interpretation of some cases, because human introductions to these islands have also been recorded, such as the European pond turtle *Emys orbicularis* [46, 47]. Also, the old links between Sardinia plus Corsica and the nearby mainland might have been overwritten by the probably more frequent and more recent links with Sicily. The latter loose biogeographic cohesiveness is only reflected by the shallowly divergent genetic lineages, indicating recent exchange between both, e.g. as shown for the Italian wall lizard *Podarcis sicula* [43, 44]. Furthermore, the biogeographic cohesiveness between Corsica and Tuscany is more pronounced than that of Sardinia and Tuscany, which is easily explained by the geographic location of Sardinia, which could exchange with the mainland via Corsica [48]. However, numerous studies have emphasised that Sardinian endemics are frequently young, and clearly of European origin [40, 49–56], hereby underlining the importance of a pathway via Corsica, or even direct immigration from the Italian mainland.

The strong phylogeographic isolation of Sicily appears at first glance unexpected when its present geographic location is considered, separated from Calabria only by the 3-km-wide Strait of Messina (Fig. 2). However, prior to the Pleistocene, the island was more isolated than today (Fig. 1a, c). During the Messinian Salinity Crisis (Miocene-Pliocene boundary), Sicily was connected with Calabria and Tunisia [25, 57], allowing an influx of African biota. In addition, low glacial sea levels repeatedly exposed large areas between Sicily and Tunisia (Fig. 1d), which served as stepping stones to facilitate later exchange [58]. Due to this geological history, Sicily has a high level of biogeographic distinctiveness compared to mainland Italy, exemplified by several island endemics, such as the Sicilian pond turtle *Emys trinacris* [47]), and Sicily is often more similar to the geographically more distant Tunisia than to nearby Calabria [4]. The similarities between Sicily and Calabria are relatively low for older and younger genetic lineages, underlining that the exchange between these regions was for long periods rather restricted, even after the final formation of Calabria at a time when they were close geographic neighbours. Furthermore, because Sicily is not orographically divided into several parts, a strong internal biogeographic structure as seen in many parts of peninsular Italy is absent, apart from some moderate differentiations, mostly in aquatic and semi-aquatic species linked to different water catchments (e.g. *Emys trinacris* [59]).

Answering our third starting question, we found that the genetic structure of the biota of peninsular Italy is highly complex and in accordance with the refugia-within-refugia model of Gómez & Lunt [8], i.e., several geographically distinct micro-refuges exist within the classical refugial centres [5]. At least some of the lineages from such micro-refuges have originated prior to the Pleistocene [60]. Such lineages have survived in different glacial micro-refugia but did not necessarily originate there. Perhaps they only retreated to these areas secondarily, during glacial cold phases, as might be the case in the Italian newt *Lissotriton italicus* [61] and the Italian wall lizard *Podarcis sicula* [44]. During the Pliocene, most of southern Italy was divided into different islands (due to higher sea levels), matching roughly the areas of Calabria, the Salentine Peninsula (the “heel” of southern Apulia), and Monte Gargano (the “spur” of Italy). Major parts of the central and northern Italian Peninsula were submerged (Fig. 1c, [3]), explaining why the most divergent, i.e. oldest, genetic lineages of many species that are today widespread in mainland Italy are detected in southern Italy.

Calabria comprised three separate islands during the Pliocene (Fig. 1c) and before (today represented by the three areas I, K and L). The most ancient genetic lineages of peninsular Italy are often found in this region, with one of the most striking examples being the Italian newt *Lissotriton italicus* [61]. Consequently, the entire Calabrian region is genetically distinct from the remaining Italian areas, reflecting the long-lasting isolation of this region [3, 12, 14]. However, only the two southern areas of Calabria (K, L) form a well-distinguished biogeographic region, whereas northern Calabria (I) represents a link to the more northern areas. This could indicate that the orographic division of Calabria still has a strong influence on the faunal composition of mainland Italy. If so, northern Calabria would represent the source (initial leading edge) of the northwards range expansions during the late Pliocene or the Pleistocene interglacials, whereas the two southern areas formed the rear edge and did not contribute to the northwards range expansions (cf. [62]).

The history and role of the Salentine Peninsula (H) is considerably different. This region was a single island during the Pliocene and before (Fig. 1a–c). Later, the Salentine Peninsula was linked to mainland Italy via the emerging Murge region (G). This is in line with the hypothesis that just one major pre-Pleistocene centre of faunal differentiation exists in this region, and that it became a major source of dispersal, influencing major parts of Central Italy. However, our results based on all lineages also reveal southern and central Apulia (G, H) as genetically more distinct than more northern regions, indicating more northern centres of differentiation during the Pleistocene, e.g., in the area of the paleo-island Gargano [26, 63], see below. Nevertheless, some of the distinctiveness of south-eastern Italy might also be due to Pleistocene immigration from the Balkan Peninsula (i.e. trans-Ionian dispersal), facilitated by the reduced size of the Adriatic Sea, or even a Quaternary land bridge (Fig. 1d; [64]). Some of these colonising organisms later became extinct in Italy, e.g. the toad *Pelobates syriacus* [64], while others still remain, such as several species of darkling beetles (Tenebrionidae) [65], the moth species *Erannis ankeraria* [66] and *Charanyca apfelbecki*, as well as the mountain butterfly *Coenonympha rhodopensis* [67].

Our results indicate that the diversification of much of the extant flora and fauna of peninsular Italy probably predates the Pleistocene and that main centres of differentiation were southern Calabria and southern Apulia. However, a number of younger differentiation centres might have been located north of that, especially in the Gargano area (E), the southern Tyrrhenian area (F) and northern Calabria (I). It is likely that these more northern centres shaped the range expansions and retractions during the Pleistocene climatic cycles, simultaneously reinforcing the genetic uniqueness of the more southern Italian areas.

The populations from the Po Plain and the two adjoining peninsular areas B and C form a single faunal unit. This applies to older and younger genetic lineages as well as to species composition. Importantly, these areas (i.e. A–C) are more strongly distinguished from southern Italy than would be expected from their location and the young geological age of the Po Plain, that originated in the Pleistocene (Fig. 1c, d; [12]). This can partly be explained by Pleistocene survival of cold-resistant taxa *in situ*. Repeated immigration from the Balkan Peninsula also contributed to the observed distinctiveness.

The Apennines, dividing peninsular Italy longitudinally from north to south, apparently had only a minor influence on the biogeographic structure of the peninsula. Thus, pairs of areas located at the same latitude but on different sides of this mountain chain (i.e., B and C; D and E) are always nearest neighbours in all cladograms based on the geographic distribution of older and younger lineages. Most of the few cases of differentiated lineages east and west of the Apennines refer to semi-aquatic species, such as the European pond turtle *Emys orbicularis* [46] or the barred grass snake *Natrix helvetica* [68]. For such taxa, the watershed formed by the Apennine chain seems to impede dispersal, fostering segregation. A similar east-west divergence (with some exceptions) is also known for the terrestrial Italian wall lizard *Podarcis siculus* [43, 44]. In general, however, the Apennines do not constitute an important biogeographic divide.

## Conclusions

The present study confirms on the meta-scale what had emerged in individual studies of smaller species´ assemblages: The Italian Peninsula and the neighbouring large islands Corsica, Sardinia, and Sicily display pronounced phylogeographic diversification patterns. For many species, the geographic density of different lineages, and sometimes the age of the splits separating lineages, are remarkably high in our study region. These characteristics are often more pronounced than the geographic complexity and ages observed for the Iberian and the Balkan peninsulas, and hence contradict the previous assumption that a relatively simple structure and shallow level of differentiation exists in Italy, due to its small size [2]. On the contrary, we conclude that the complex and diverse geological history of Italy, including strong tectonic activity, shifting (micro)plates, the submergence of major parts of the current peninsula, the subsequent formation of numerous islands, as well as the Pleistocene climatic cycles (Fig. 1) have fostered the evolution of the highly complex biogeographic patterns observed today and an unexpected richness in biodiversity. The long-lasting isolation of Sicily as well as Sardinia plus Corsica has produced old endemic biota, which only recently (i.e., during the Pleistocene) have moderately interacted with the neighbouring mainland regions. The vanished Pliocene islands in the Calabrian and southern Apulian areas are the origin of other endemic biota, which only recently expanded northwards, with further differentiations. Continuous immigration from the Balkan Peninsula to northern Italy, combined with subsequent ice age survival *in situ*, is most likely responsible for the unexpectedly high biogeographic distinctiveness of this region.

## Supplementary Information

**Additional file 1: Appendix S1.** Overview of all studies analysed with main phylogeographic patterns.

**Additional file 2: Appendix S2.** (a) Number of genetic lineages within 17 Italian biogeographic areas used for the present study. (b) Geographic distribution of all lineages over the 17 Italian biogeographic areas. (c) Sørensen dissimilarity matrix between 17 Italian biogeographic regions based on 213 and 258 genetic lineages, respectively.

**Additional file 3: Appendix S3.** (A1) Unrooted neighbour joining cluster analyses (Sørensen similarities) of vertebrate (Fig. A1) and plant species (Fig. A2).

## Data Availability

Articles from which data were used in our analyses are compiled in an electronic appendix.
