# Supplementary material for: Biogeography of Italy revisited: genetic lineages confirm major phylogeographic patterns and a pre-Pleistocene origin of its biota
Source: Front Zool. 2021 Jun 29;18:34. doi: 10.1186/s12983-021-00418-9 (PMC8240252; doi:10.1186/s12983-021-00418-9)

Appendix S4

**Biogeography of Italy revisited: Genetic lineages confirm major phylogeographic patterns and a “museum of the Neogene”**

Thomas Schmitt, Uwe Fritz, Massimo Delfino, Werner Ulrich, Jan Christian Habel

Figure A1. Neighbour joining cluster analyses (Soerensen dissimilarities) of the occurrence of 150 deeply divergent level I and shallower divergent level II genetic vertebrate lineages. Numbers show the percentage of bootstrapped trees (1,000 replicates) corroborating the focal split. Colours link areas consistently clustering together. Rectangles mark the nearest clusters of Sicily. Order of areas best matches the latitudinal mainland gradient.


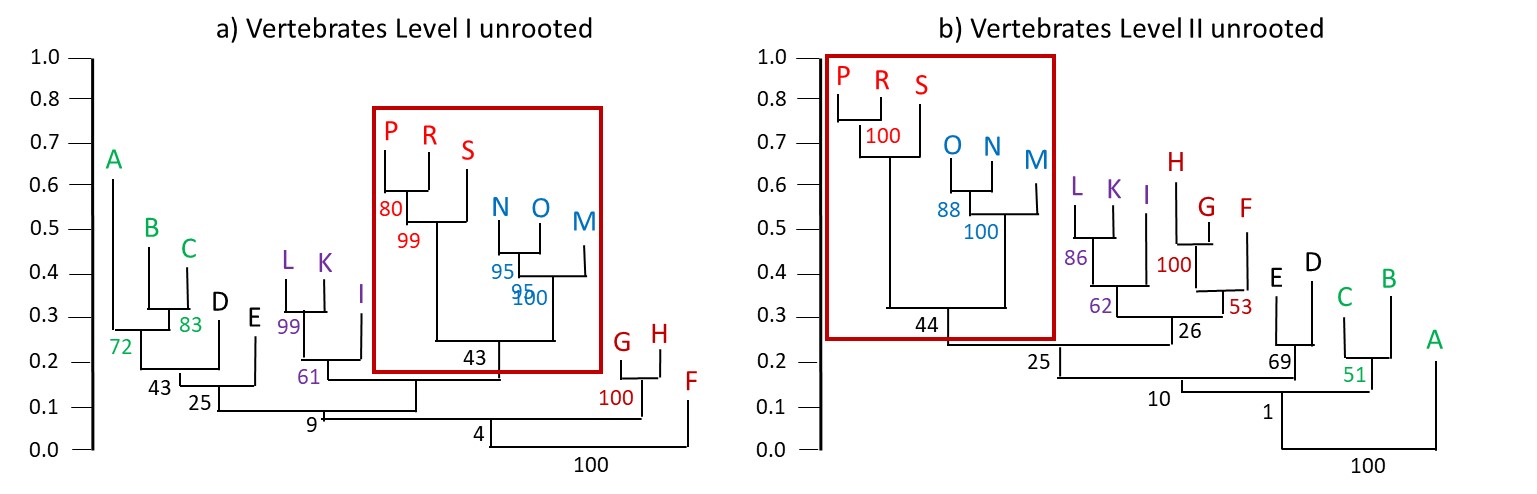


Figure A2. Unrooted neighbour joining cluster analysis (Soerensen similarities) of the occurrence of 42 genetic lineages classified into a deeply divergent genetic level within 13 plant species among 17 areas (A–S) as defined in Fig. 1. Numbers show the percentage of bootstrapped trees (1,000 replicates) corroborating the focal split. Colours link areas consistently clustering together. Order of areas best matches the latitudinal mainland gradient.


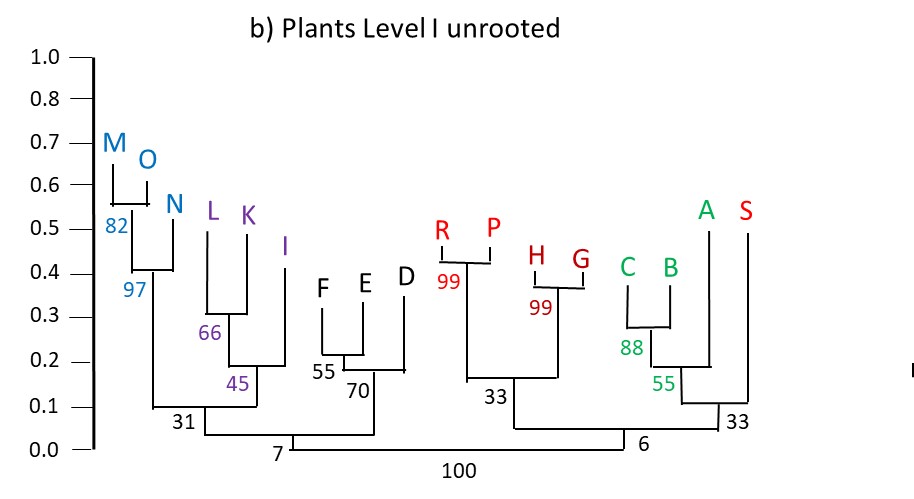

Supplement: Supplementary file 3 — Additional file 3: Appendix S3. (A1) Unrooted neighbour joining cluster analyses (Sørensen similarities) of vertebrate (Fig. A1) and plant species (Fig. A2). [file 12983_2021_418_MOESM3_ESM.docx]
